# Mass-Production and Characterizations of Polyvinyl Alcohol/Sodium Alginate/Graphene Porous Nanofiber Membranes Using Needleless Dynamic Linear Electrospinning

**DOI:** 10.3390/polym10101167

**Published:** 2018-10-19

**Authors:** Ting-Ting Li, Mengxue Yan, Wenting Xu, Bing-Chiuan Shiu, Ching-Wen Lou, Jia-Horng Lin

**Affiliations:** 1 Innovation Platform of Intelligent and Energy-Saving Textiles, School of Textiles, Tianjin Polytechnic University, Tianjin 300387, China; litingting_85@163.com (T.-T.L.); 13032210827@163.com (M.Y.); 15222178020@163.com (W.X.); 2 Tianjin and Education Ministry Key Laboratory of Advanced Textile Composite Materials, Tianjin Polytechnic University, Tianjin 300387, China; 3 Laboratory of Fiber Application and Manufacturing, Department of Fiber and Composite Materials, Feng Chia University, Taichung 40724, Taiwan; toyysbk@gmail.com; 4 Department of Bioinformatics and Medical Engineering, Asia University, Taichung 41354, Taiwan; 5 Department of Chemical Engineering and Materials, Ocean College, Minjiang University, Fuzhou 350108, China; 6 College of Textile and Clothing, Qingdao University, Qingdao 266071, China; 7 School of Chinese Medicine, China Medical University, Taichung 40402, Taiwan; 8 Department of Fashion Design, Asia University, Taichung 41354, Taiwan

**Keywords:** needleless electrospinning, linear electrode, graphene (Gr), nanofibers, conductivity

## Abstract

The aim of this study was to investigate the feasibility of large-scale preparation of porous polyvinyl alcohol/sodium alginate/graphene (Gr) (Gr-AP) nanofiber membranes using a copper wire needleless dynamic linear electrode electrospinning machine. Furthermore, the effects of Gr concentrations (0, 0.0375, 0.075, 0.25, 0.5, and 0.75 wt.%) on the morphology, electrical, hydrophilicity and thermal properties were evaluated. Results indicate that the dynamic linear electrospun Gr-AP membranes have a high yield of 1.25 g/h and are composed of porous finer nanofibers with a diameter of 141 ± 31 nm. Gr improved the morphology, homogeneity, hydrophobicity and thermal stability of Gr-AP nanofiber membranes. The critical conductive threshold is 0.075 wt.% for Gr, which provides the nanofiber membranes with an even distribution of diameter, an optimal conductivity, good hydrophilicity, appropriate specific surface area and optimal thermal stability. Therefore, needleless dynamic linear electrospinning is beneficial to produce high quality Gr-AP porous nanofiber membranes, and the optimal parameters can be used in artificial nerve conduits and serve as a valuable reference for mass production of nanofiber membranes.

## 1. Introduction

The incidence of nerve injuries has risen year by year as a result of the prosperity of public transportation and the increase in gross national income [1]. Considering long-term disability and poor surgery outcomes, peripheral nerve injury presents a substantial challenge to reconstructive surgeons, and autologous nerve grafts is thus a commonly employed measure [2]. Up until now, diverse natural and synthetic polymer biomaterials have been used for the development of scaffolds in the peripheral nerve tissue engineering field [3,4,5,6,7,8].

Electrospun nanofibers have characteristics of high porosity, high specific surface area, and good mechanical properties, which qualify for their use in environmental monitoring, healthcare, environmental protection, and electronics [9]. Electrospinning can transform suitable materials into nanofiber scaffolds, which can effectively substitute neural bridge connection [10,11,12,13]. Moreover, sodium alginate (SA) has good biocompatibility, biodegradability and has commonly been used as an artificial substitute of peripheral nerve tissues. However, due to its high viscosity, the SA solution cannot be electrospun into nanofibers individually, and is usually blended with polymers like PVA with good fiberizability to form nanofibers [14,15,16]. In addition, graphene (Gr) has good mechanical, electrical, thermal, optical properties, and a high specific surface area. Some scholars have used Gr to modify the properties of tissue engineering PVA/alginate scaffolds, thereby obtaining extraordinary electric conductivity and accelerating cell proliferation [17,18,19,20,21,22].

Despite of a competitive potential with advantages of efficiency, a lower production cost, manageable processes, and zero pollution, electrospinning has never been applied to industrialization. Traditional needle electrospinning fails to proceed to efficient production, and the issue of needle blocking also restricts its development. Likewise, needleless electrospinning also has drawbacks and falls short of the demands of the market [23,24,25,26,27,28,29]. For example, static bubble spinning produces fibers with a diameter dependent on the bubble size, and the fibers diameters are commonly uneven [30]. Similarly, using a dynamic rotating cylinder as the spinning nozzle produces a great range in the diameters of nanofibers [31,32]. In addition, when using PVA in the electrospinning materials, cone metallic coil electrospinning has a yield of 2.5 g/h, which is thirteen times higher than conventional single needle electrospinning (less than 0.3 g/h) [33,34,35,36] and the nanofibers have a lower diameter than needle electrospinning. For example, Golafshan et al. used needle electrospinning to form Gr-AP nanofiber stents that were suitable for nerve engineering and the nanofibers had a diameter above 300 nm. However, its low yield cannot satisfy market demand, and the discontinuous electrospinning process is not suitable for mass production [19]. In our previous study, using the cylindrical dynamic linear electrode as the spinning nozzle can achieve continuous electrospinning, with finer diameter nanofibers and a higher yield of nanofiber membranes [37].

In order to improve the electrospinning process to the greatest extent, increase product yields, and produce composite nanofibers with a high specific surface area and excellent properties, this study uses a custom-made copper wire needleless dynamic linear electrode electrospinning machine [38] (patent #2015208045157) to make Gr-AP porous nanofiber membranes out of PVA, SA, and Gr. The optimal Gr content, collection distance, and electrospinning voltage are adjusted based on the morphology and diameter distribution of the nanofibers. Moreover, the influence of the presence of Gr is studied in terms of electrical properties, hydrophilicity, and thermal stability of the nanofiber membranes. This study achieves its goal of the preparation of Gr-AP nanofiber membranes with a high production yield. This process can serve as a valuable stepping stone for the newly and highly efficient manufacture of nanofiber membranes for man-made neutral conduits.

## 2. Experimental Section

### 2.1. Materials

**Figure 1:**
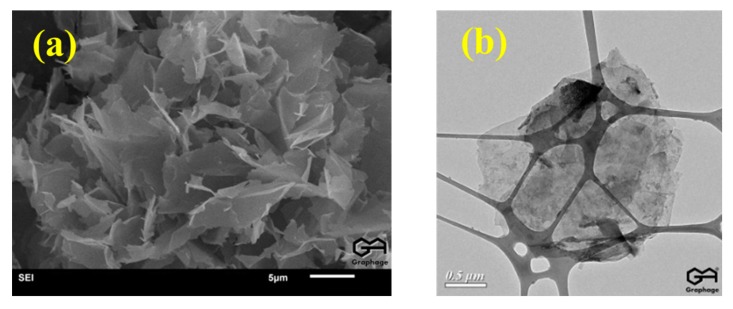
SEM and TEM observations of graphene.

Polyvinyl alcohol (PVA, M_w_ = 84,000–89,000) was purchased from Changchun Chemical, Changchun, China. Sodium alginate (SA, purity = 90%) and polyvinylpyrrolidone (PVP, K13-18, M_r_ = 10,000) were purchased from Shanghai Macklin Biochemical, Shanghai, China. P-ML20Multi-layered graphene (Gr) was provided from Enerage, Taiwan. The average thickness of Gr is 50–100 nm (Figure 1), specific surface area is <50 m^2^/g, and the conductivity is >700 S/m. Deionized water was used in this study.

### 2.2. Formulation of Gr and Gr-AP Suspensions

Gr was firstly dispersed into 1% PVP dispersing agent, and then underwent ultrasonic oscillation at 40 kHz for 2 h to obtain the stabilized Gr solutions. Moreover, 13.5 g of PVA was added to Gr solutions and then stirred with a magnetic heating stirrer at 90 °C for 2 h, formulating PVA/Gr suspensions with diverse concentrations. Finally, 2% of SA solution (20 mL) was added to the PVA/Gr suspension and then stirred at 60 °C for 2 h, evenly forming PVA/SA/Gr suspensions with different Gr contents. The concentrations of Gr in PVA/SA/Gr suspensions were 0, 0.0375, 0.075, 0.25, 0.5, and 0.75 wt.% respectively. The viscosity and conductivity of PVA/SA/Gr suspensions are shown in Table 1.

### 2.3. Preparation of Linear Electrospinning Nanofiber Membranes

**Figure 2:**
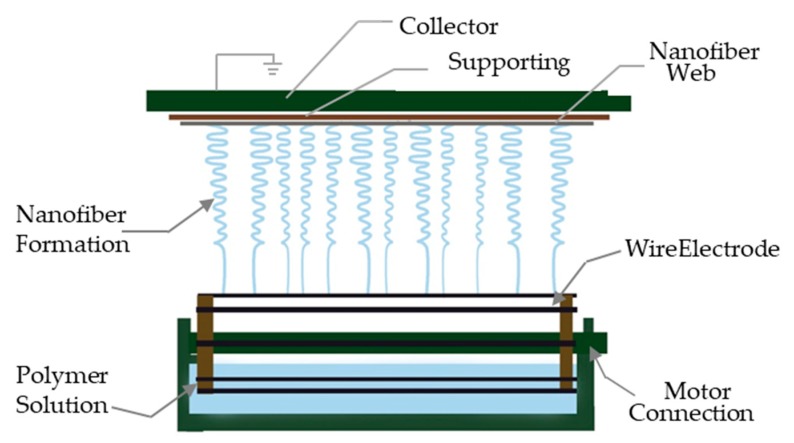
Schematic diagram of the needleless linear electrospinning.

**Figure 3:**
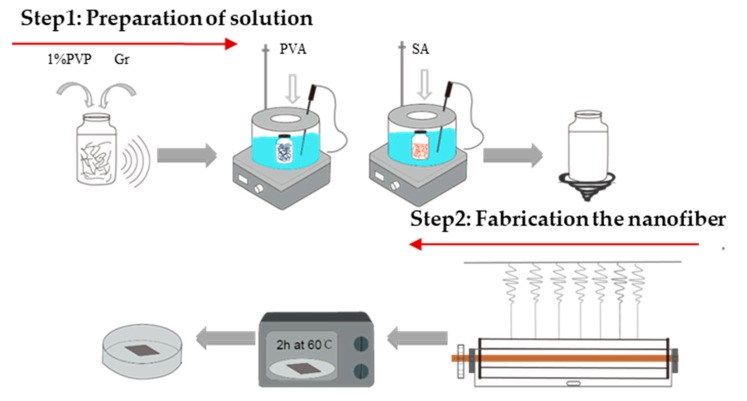
Process of the preparation of linear electrospinning nanofiber membranes.

PVA/SA/Gr suspensions with different Gr contents were electrospun into AP and Gr-AP nanofiber membranes for 5 h using a custom-made copper wire needleless dynamic linear electrode electrospinning machine (Figure 2). The collection distance was set as 25, 27.5 and 30 cm, respectively. Additionally, the voltage was set as 70, 75 and 80 kV, respectively. The membranes were dried in an oven at 60 °C for 2 h and evaluated in terms of the morphology and property evaluations. The whole process of the preparation of linear electrospinning nanofiber membranes are shown in Figure 3. The electrospinning principle was as follows: Four 0.8-mm-diameter copper wires are used as the linear electrode that is connected to a high voltage and immersed in a suspension. A motor rotates the copper linear electrodes, ensuring a complete adhesion of the electrospinning solution. The electrostatic force is strengthened when the linear electrode approaches the collector, and the spherical liquid over the electrode eventually forms a Taylor cone. The triggered jets and the electrical jets become fine nanofibers because of the drawing force, during which the solvent evaporates, and the nanofibers can deposit onto the collector irregularly to form a nanofiber membrane.

### 2.4. Measurements and Characterizations

The morphology of AP and Gr-AP nanofiber membranes was observed using scanning electron microscopy (SEM, TM3030, HITACHI, Tokyo, Japan). The SEM images were analyzed using Image-Pro Plus 6.0 software. A bundle of 100 nanofibers per image were used to measure the diameter of the nanofibers, and Origin was used to plot the diameter distribution and to compute the standard deviations. The functional groups of nanofiber membranes were analyzed using a NICOLET iS10 FT-IR spectrometer (Thermo Fisher Scientific, Waltham, MA, USA), and the surface resistivity of nanofiber membranes was measured using surface resistance instrument (RT-1000, OHM-STAT, Static Solutions Inc, Hudson, NY, USA), examining the influence of Gr content on the conductive threshold. The crystalline structure of the AP and Gr-AP nanofiber membranes was characterized by X-ray diffraction (D8 DISCOVER, BRUKER, Billerica, MA, USA) with CuKa radiation (λ = 1.5406 Å). The wettability and hydrophilicity of nanofiber membranes were characterized by the water contact angle measured using a surface contact angle instrument (JC2000DM, Shanghai Zhongchen Digital Technic Apparatus, Shanghai, China) and Image-Pro Plus 6.0 software. The contact angle between the droplet at first, second and surface of samples was measured five times to calculate the mean. The melting pattern of nanofiber membranes was measured using a differential scanning calorimetry (DSC200F3, NETZSCH, Bavaria, Germany) with nitrogen as the shielding gas. Samples of 5–10 mg were heated from the room temperature to 300 °C at 10 °C/min. The thermal stability of nanofiber membranes was measured using a thermogravimetric analyzer (TG 209F3, NETZSCH, Bavaria, Germany) with nitrogen as the shielding gas. Samples of 5–10 mg were heated from the room temperature to 700 °C at a rate of 10 °C/min. The adsorption and surface area were characterized using a Quantachrome Autosorb instrument (IQ-C, KANGTA, Boynton Beach, FL, USA) with nitrogen as the adsorbate. The Barret-Joyner-Halenda (BJH) model was used to determine the distribution of mesopores.

## 3. Results and Discussion

### 3.1. Effect of Gr Content, Collection Distance and Voltage on Morphology and Diameter of Gr-AP Nanofiber Membranes

**Figure 4:**
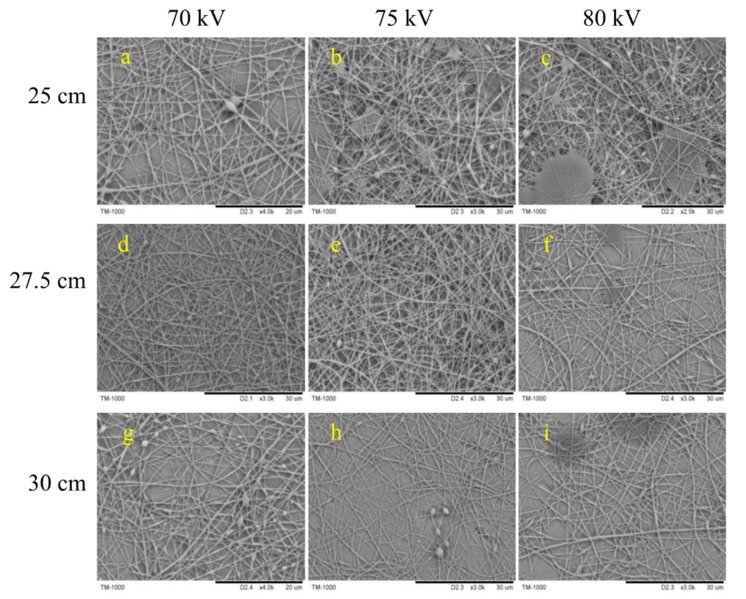
SEM images of Gr-AP nanofiber membranes containing 0.0375 wt.% of Gr. The collection distance is 25, 27.5, and 30 cm, and the electrospinning voltage is 70, 75, and 80 kV.

**Figure 5:**
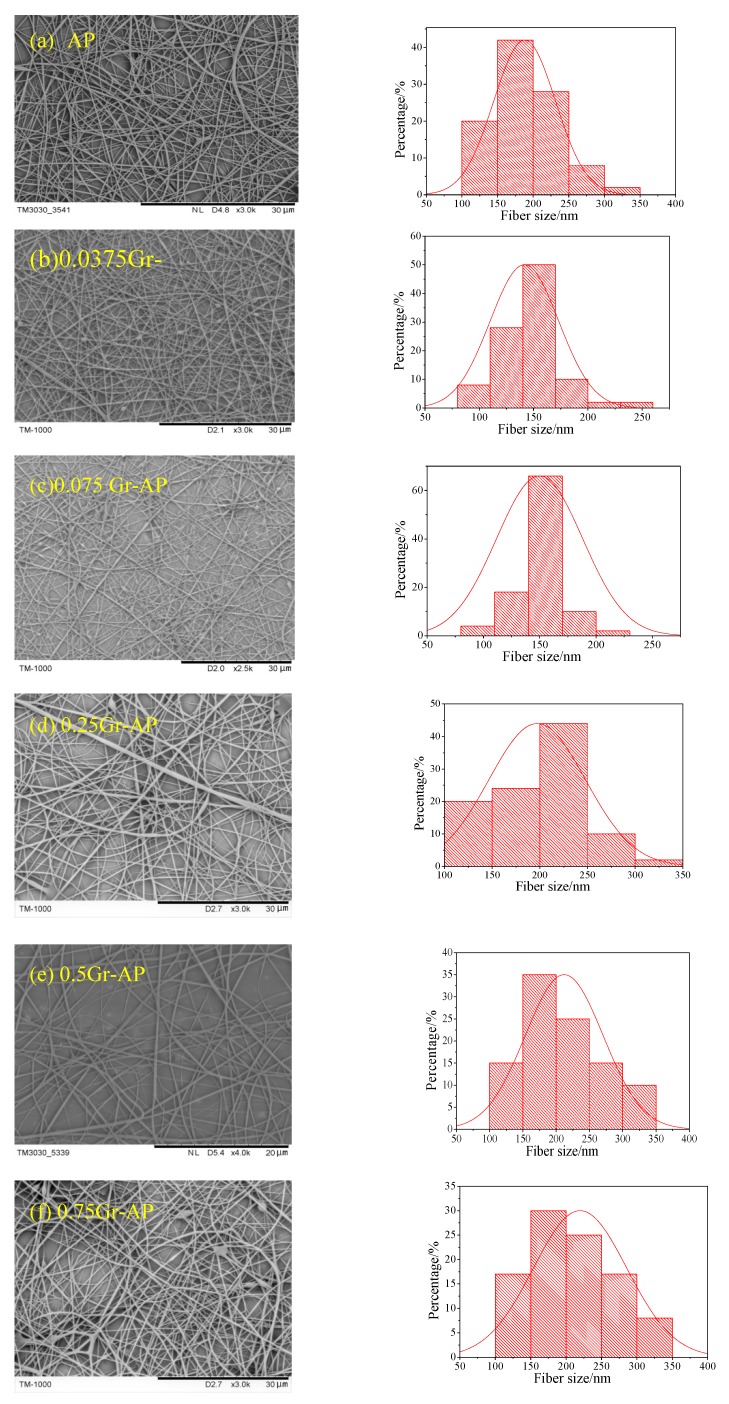
SEM images and fiber diameter distributions of AP-Gr membranes with different Gr contents.

Figure 4 shows the SEM images of electrospun Gr-AP nanofiber membranes containing 0.0375 wt.% (0.0375Gr-AP). A large number of droplets were adsorbed over the surface of nanofibers when the collection distance is small, and the nanofiber membrane exhibit many beads as seen in Figure 4a–c. As the collection distance increases, the morphology of the nanofibers improved. This is because the attenuating attraction of static electricity allows a longer time for droplets to be drawn, which gives the solvent more time to evaporate. However, an excessive collection distance debilitates the electrical fields and produces unstable electrospinning jets, which render nanofibers with a larger diameter and an uneven surface, as shown in Figure 4g. The nanofibers morphology is related to the applied field (*E*∞ = *V/D*) according to Coulomb’s law, and the Gr-AP nanofiber membranes present optimal microstructure within an appropriate distance (*D*) and voltage (*V*). As seen in Figure 4, the optimal membrane morphology occurs at 27.5 cm (*D*) and 70 kV (*V*). The regulation of structure of Gr-AP nanofiber membranes with other Gr contents was consistent with that of 0.0375 Gr-AP nanofiber membranes. The morphology and diameter distribution of Gr-AP nanofiber membranes at optimal distance and voltage are displayed in Figure 5. It can be seen in Figure 5 that at the optimal distance and voltage, all the Gr-AP suspensions with different Gr content can be successfully electrospun into nanofibers and that the nanofibers exhibited a smooth surface with few beads and uniform fiber diameter distribution. The mass production made a prominent improvement with a yield of Gr-AP membrane reaching 1.25 g/h, which is higher than needle electrospun Gr-AP nanofiber membranes indicated by Golafshan et al. [19].

**Figure 6:**
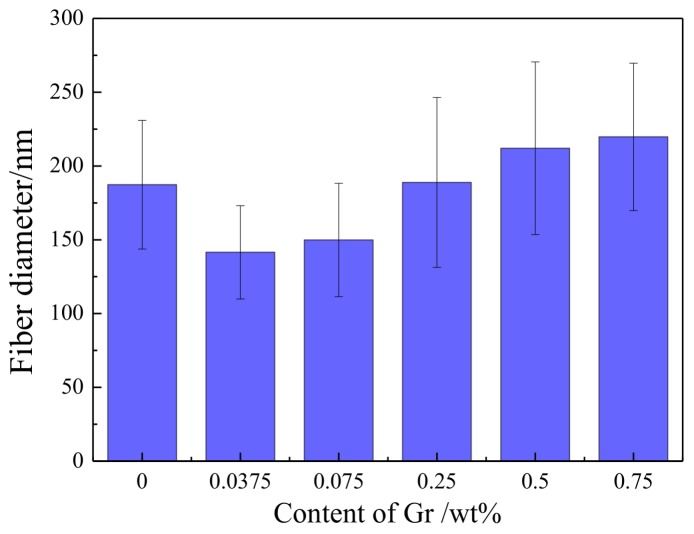
Diameter of nanofibers of Gr-AP nanofiber membranes as related to the Gr content.

Figure 6 shows the average diameter and standard deviation (SD) of Gr-AP nanofiber membranes as related to different Gr concentrations. With the increase of Gr content, the diameter of nanofibers proportionally increases, and the nanofibers also have an increasingly uneven surface. The viscosity and conductivity of PVA/SA/Gr suspensions is related to the fiber diameter according to the electro hydrodynamic model. The viscosity of PVA/SA/Gr mixture increased with the addition of Gr as seen in Table 1, and higher voltage was required to obtain the stretched jet. While the applied electric field (*V*/*D*) is constant, the spinning process is more difficult and as a result, and the mean diameter of the fibers increases [39]. At less than 0.25 wt.% of Gr, the finest Gr-AP nanofibers of 141 ± 31 nm, is much smaller than that of Gr-AP membrane produced by the needle electrospining (296 ± 40 nm) proposed by Golafshan et al. [19], and the overall nanofibers have good morphology and even diameters. The smaller diameter of nanofibers is due to multiple factors, a small amount of Gr improves the conductivity of the suspension, rendering droplets over the linear electrodes with considerable electric charges. Simultaneously, the repulsion between the electric charges decreases the surface tension of the liquid, which makes the nanofibers subject to splitting during electrospinning. When surpassing 0.25 wt.%, an excessive amount of Gr aggregate and generate nodes over the nanofibers. Subsequently, the conductivity of the suspension increases, which is detrimental to the electrospinning due to whipping instability, and results in coarser and more uneven diameter of fibers [40].

### 3.2. Far Infrared Ray Spectrum of Gr-AP Nanofiber Membranes

**Figure 7:**
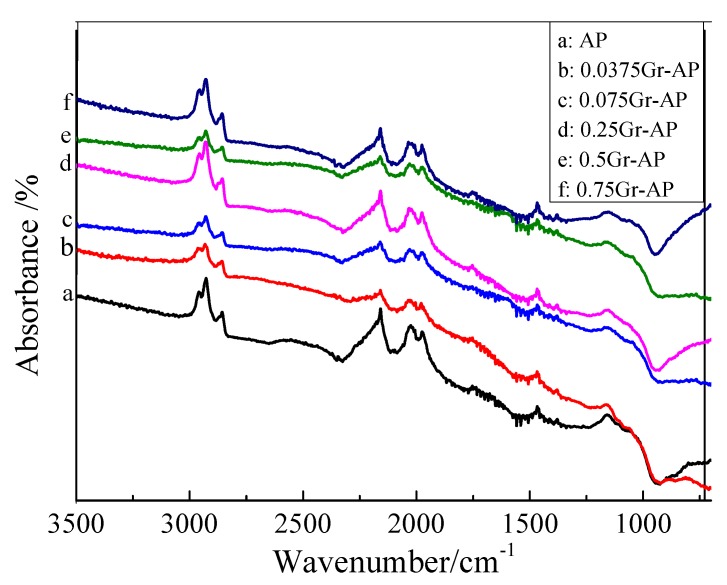
FIR spectrum of Gr-AP nanofiber membranes as related to the Gr content.

Figure 7 shows that Gr-AP and AP nanofiber membranes have comparable spectra of absorbance. The presence of characteristic peak at 2850 cm^−1^ indicated the stretching vibration between the C–H and O–H from PVA, SA, and the residual water. The absorption peak at 2210.9 cm^−1^ corresponded to C=O for –COOH. The peak of the stretching vibration of C=C was presented at wave numbers of 1992.2 cm^−1^, while the peak of the stretching vibration of C=O for C–O–C was presented at wave numbers of 1119.8 cm^−1^ [41]. The corresponding characteristic peaks of the functional groups of AP and Gr-AP nanofiber membranes were presented, suggesting that Gr and PVA/SA suspensions were successfully mixed.

### 3.3. X-Ray Diffraction of Gr-AP Nanofiber Membranes

**Figure 8:**
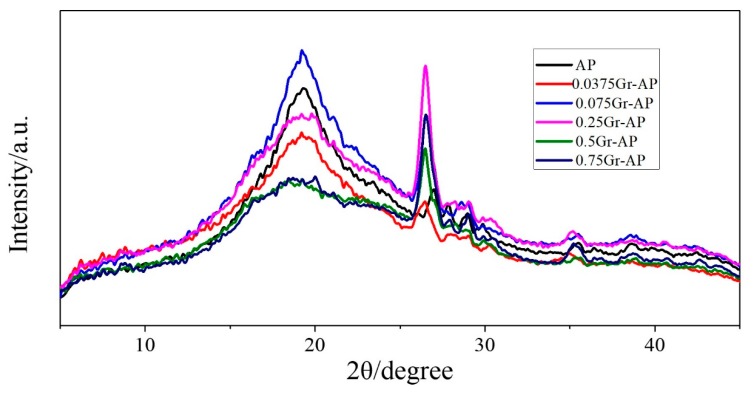
XRD pattern of Gr-AP nanofiber membranes as related to the Gr content.

In order to identify the existence of Gr in the electrospun nanofibers, XRD results are displayed in Figure 8. AP membrane exhibited a broad diffraction peak at 2θ = 19.6°, indicating the interaction and blending between SA and PVA. In addition, this broad peak is because hydrogen bond interactions between –OH and –COOH from SA and between –OH groups formed non-crystalline nanofibers [19]. Furthermore, a sharp diffraction peak occurred at 2θ = 27° for AP membrane. After Gr addition, these two diffraction peaks shifted towards to the left (2θ = 19.1° and 2θ = 26.5°), and the diffraction intensity also changed. This is because Gr addition caused a larger lattice constant [19] which confirms the existence of Gr.

### 3.4. Electrical Properties of Gr-AP Nanofiber Membranes

**Figure 9:**
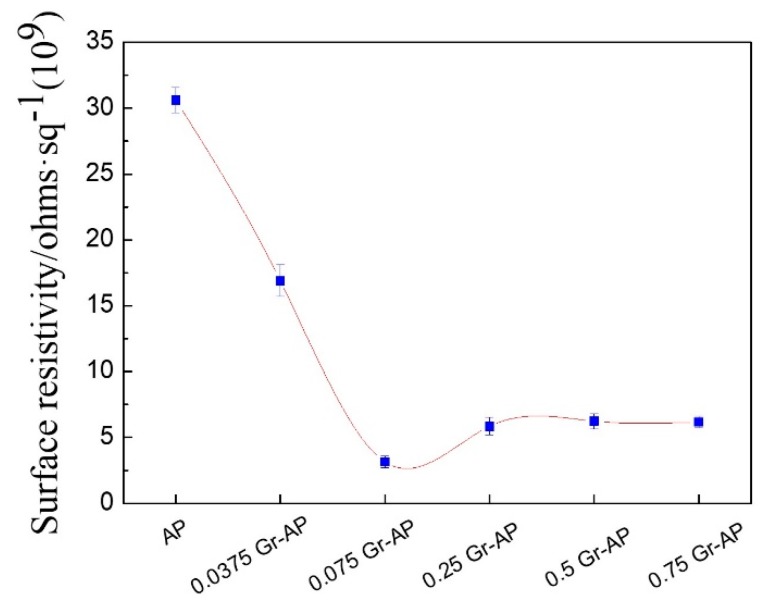
Surface resistivity of Gr-AP nanofiber membranes as related to the Gr content.

The surface resistivity of Gr-AP nanofiber membranes as related to the Gr content is shown in Figure 9. Compared to AP membrane, Gr significantly decreased the surface resistance and improved the conductivity of membranes. With the addition of Gr, the surface resistivity first decreases sharply (0–0.075 wt.% Gr) and then increases (0.075–0.25 wt.% Gr) and slightly decreases (0.25–0.75 wt.% Gr). When Gr content is 0.075 wt.%, the surface resistivity remarkably decreased to 3.13 × 10^9^. This is because a low Gr content allows Gr to form broad conductive paths in a network that accelerates electric conduction. As Gr increases continuously (>0.075 wt.%), the surface resistance increases first and then decreases slightly, while the conductivity decreases first and then increases correspondingly. This result is due to the aggregation of Gr in excessive amounts, which in turn produces impedance paths. In addition, nanofiber composites demonstrate a percolation phenomenon, which involves a balanced critical concentration of the interacting materials. At a percolation threshold, the network that transmits electrons is dependent on the coagulation of fillers [42]. It can be surmised that Gr has a critical concentration of 0.075 wt.% as an electrical percolation threshold, and the adjacent Gr forms a tunneling effect [43]. As a result, the conductivity of Gr-AP nanofiber membranes containing 0.075 wt.% Gr or more is ten times that of AP nanofiber membranes.

### 3.5. Water Contact Angle of Gr-AP Nanofiber Membranes

**Figure 10:**
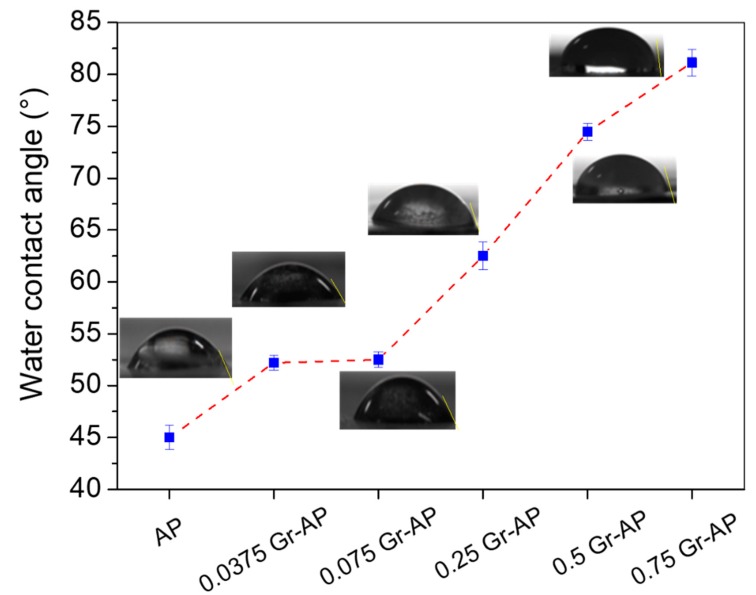
Water contact angle of Gr-AP nanofiber membranes as related to Gr content.

Water contact angle was applied to investigate the hydrophilic properties of Gr-AP nanofiber membranes (Figure 10). Due to hydrophilic nature, AP membranes revealed low hydrophilic properties at a water contact angle of 45° ± 1.6°. Moreover, water contact angle enhanced significantly in relation to Gr content, demonstrating superior hydrophobicity. For the Gr-AP nanofiber membrane containing 0.75 wt.% Gr, the water contact angle increased to 81.14° ± 1.3°, a factor of 20° higher than needle electrospun AP-Gr membrane at the same Gr content [19]. The presence of Gr and more compact nanofiber structure could explain the difference between them. As described in Figure 6, the fiber diameters of AP-Gr membranes formed compact structures. The increased hydrophobicity is advantageous for nerve tissue engineering applications. On the one hand, the hydrophilic scaffold has the important characteristic of a high degradation rate, which greatly decreased the mechanical property in the tissue regeneration process, and thus limits the application of fiber scaffold in nerve tissue engineering [44]. On the other hand, the hydrophobic nanofiber membrane can effectively inhibit the cell proliferation, and the hydrophobic inner shell can guarantee the resistance to fibroblasts [45,46]. Therefore, the contact angle result confirms the Gr addition can effectively enhance the hydrophobicity of fibrous membranes, which is beneficial to the nanofiber membrane applied to neural tissue engineering.

### 3.6. Thermal Properties of Gr-AP Nanofiber Membranes

**Figure 11:**
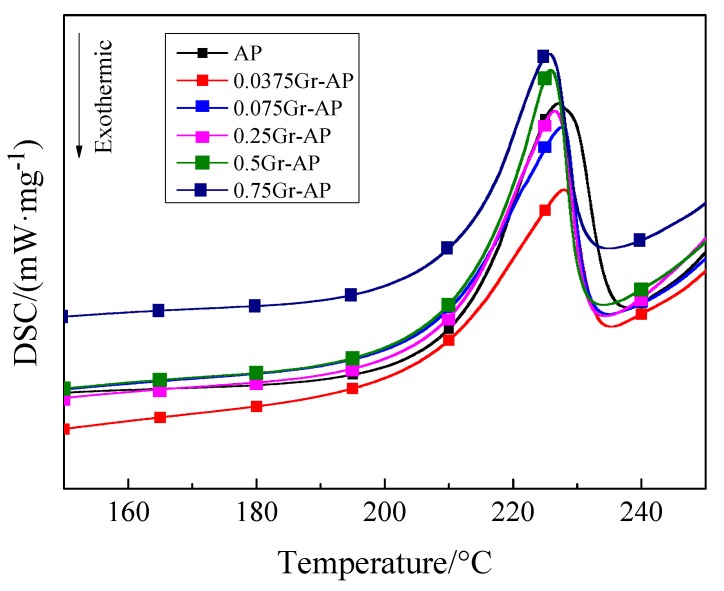
DSC curves of Gr-AP nanofiber membranes as related to the Gr content.

Figure 11 shows that DSC curves of AP and Gr-AP nanofiber membranes display similar, and both have a melting peak within 220–230 °C. The melting peak of AP nanofiber membranes was 227.1 °C, and the addition of Gr shifted the melting peak toward higher temperatures. Adversely, a Gr content that exceeds 0.075 wt.% shifted the melting peak toward lower temperatures. The results may be ascribed to the dispersion of Gr. As –COOH and –OH are generated over the surface of Gr, –OH and its oxgenous functional groups of the PVA molecular chains are hydrogen bonded, the resulting interaction adversely affects the mobility of PVA and SA molecular chains [47]. By contrast, an excessive Gr content causes agglomeration and then decreases the melting point.

**Figure 12:**
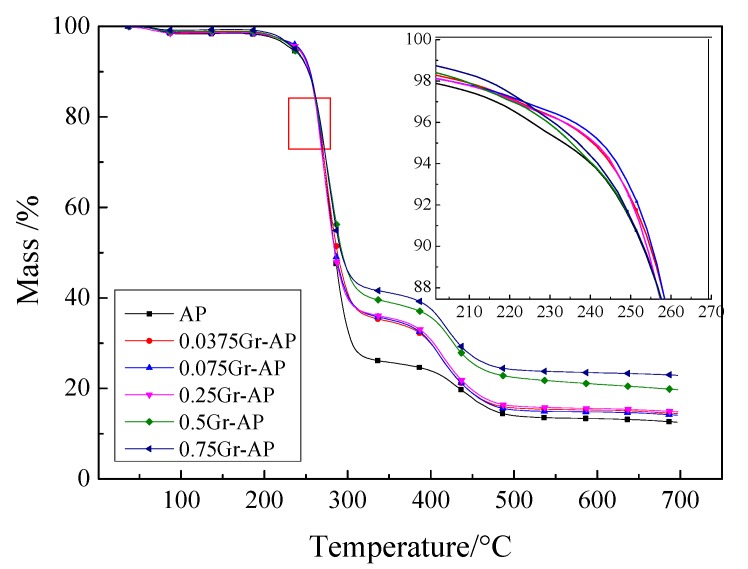
TG curves of Gr-AP nanofiber membranes as related to the Gr content.

Figure 12 shows TG curves that demonstrate three weight loss phases. The first phase occurred when the temperature was between 50 and 100 °C. It was caused by the evaporation of the residual water or the solvent, and the weight loss was relatively smaller. The second weight loss phase occurred due to the degradation peaks between 100 and 350 °C. A considerable thermal decomposition of nanofiber membranes occurred in this phase, due to the breakage of macromolecular chains of PVA and SA. Comparing the slopes, the maximum decomposition rate was also seen in this phase with a weight loss of 55–75%. The third weight loss phase occurred at 350–600 °C when the breakage of molecular chains and cyclization were rendered to PVA and SA. More molecular chains of residual polymer polyenes further broke and were converted into micromolecule polyenes, with a weight loss of 10–20% [48].

It can be found from Figure 12 that the TG curves at the first and second stages are similar regardless of the Gr content. As described in Table 2, at 90% mass of nanofiber membranes, the water in the membranes was completely evaporated, and PVA and SA started decomposing. Gr-AP nanofiber membranes had a higher decomposing temperature of 90% mass compared to AP membranes. The TG curve of Gr-AP nanofiber membranes exhibits a shallower slope than that of AP nanofiber membranes, indicating a slow degradation status. It was surmised that the presence of Gr increases the decomposition temperature of nanofiber membranes, thereby retarding the decomposition rate and improving the thermal stability. Specifically, composed of 0.075 wt.% of Gr, the Gr-AP nanofiber membranes have the highest decomposition temperature. The higher the Gr content, the more the residual mass of Gr-AP nanofiber membranes, suggesting that Gr is not easily decomposed at 700 °C.

### 3.7. BET Specific Surface Area of Gr-AP Nanofiber Membranes

**Figure 13:**
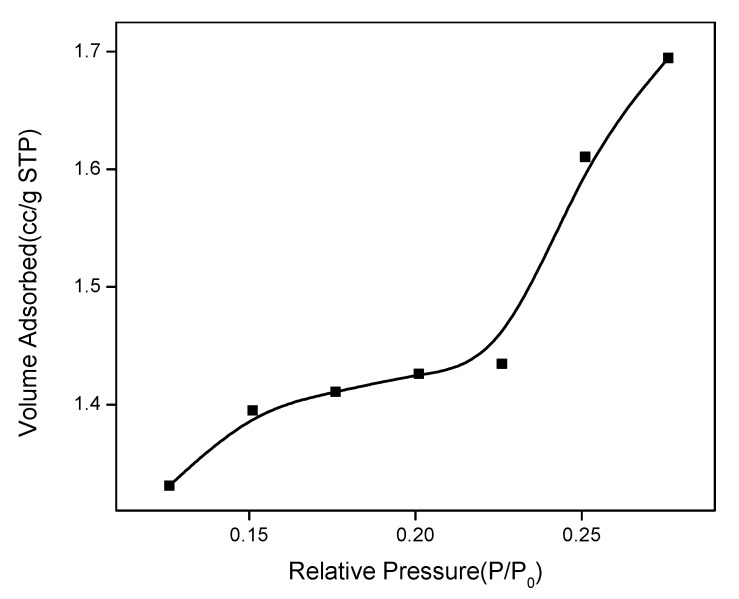
N_2_ adsorption isothermal curves of 0.075Gr-AP membrane based on the BJH model.

Porous structure of Gr-AP membrane is analyzed by BET measurement. Figure 13 shows the N_2_ adsorption isothermal curves of Gr-AP membrane. The N_2_ adsorption/desorption isothermal curve presents a rapid increase when relative vapor pressure of P/P_0_ is lower than 0.15, then an increase to an inflection point, and finally a linear increase when relative vapor pressure of P/P_0_ is higher than 0.22. This adsorption isotherm curve represents an S-type isotherm and belongs to the macropore (>50 nm) free single multilayer reversible adsorption process. The tendency is in conformity with type II adsorption behavior [49,50], indicating the macropore structure of the Gr-AP membrane. The BET result further showed that the Gr-AP nanofiber membrane has high specific surface area of 5.347 m^2^/g, and high specific surface area has relative high porosity, which is beneficial to cell attachment and growth in the nerve tissue engineering.

## 4. Conclusions

In this study, it was demonstrated that the custom-made needleless linear electrodes can successfully electrospin into porous well-formed Gr-AP nanofiber membranes. The test results show that the presence of Gr improved the properties of Gr-AP nanofiber membranes. The average diameter of Gr-AP nanofiber membranes is 141 ± 31 nm, which is 150 nm finer than that of needle electrospun nanofibers. Moreover, the achieved mass production makes progress over other methods with a yield of 1.25 g/h. When made with different Gr contents, the morphology of Gr-AP nanofiber membranes can be manipulated using different voltages with a specified collection distance. With a critical value, using Gr improves the properties of Gr-AP nanofiber membranes. Increasing Gr content from 0 to 0.075 wt.% improves the morphology and homogeneity of Gr-AP nanofiber membranes, contributing greater hydrophobicity and thermal stability than those of pure AP nanofiber membranes. When Gr content is 0.075 wt.%, the conductivity of Gr-AP nanofiber membranes remarkably changes and then stabilizes. Conversely, the Gr content that exceeds 0.075 wt.% renders uneven diameter of nanofibers and weakened thermal stability of nanofiber membranes. Hence, a Gr content of 0.075 wt.% is presumed to be the threshold when producing Gr-AP nanofiber membranes. As a result, the linear electrospinning achieved a highly efficient production of Gr-AP nanofiber membranes. This study serves as a valuable reference to prepare nanofibers for neural conduits. Furthermore, Gr-AP nanofiber membranes have high specific surface area of 5.347 m^2^/g, good hydrophobicity, good biodegradability and appropriate degradation rate, which will be used for regenerated fibrous membrane in the nerve tissue engineering.

## Figures and Tables

**Table 1:** Viscosity and conductivity of PVA/SA/Gr suspensions with different Gr content.

Gr Content (wt.%)	0	0.0375	0.075	0.25	0.50	0.75
Viscosity (±0.1 mPa·s)	331	320	340	346.2	348.9	397
Conductivity (±0.01 μS·cm^−1^)	865	795	818	858	878	900

**Table 2:** DSC and TG results of Gr-AP nanofiber membranes as related to the Gr content.

Samples	Melting Point (°C)	T90% (°C)	Mass Change at the First Stage (%)	Mass Change at the Second Stage (%)	TG Residual Mass (%)
AP	227.1	253.0	72.55	13.28	12.5
0.0375Gr-AP	227.9	254.9	63.26	20.38	14.07
0.075Gr-AP	227.7	255.3	63.89	21.16	14.42
0.25Gr-AP	226.4	254.1	62.89	20.76	14.84
0.5Gr-AP	225.8	253.3	59.88	19.30	19.70
0.75Gr-AP	225.7	253.2	58.02	18.24	22.89

## Founding

This work is supported by the National Natural Science Foundation of China (grant numbers 51503145,11702187); the Natural Science Foundation of Tianjin City (grant number 18JCQNJC03400); the Natural Science Foundation of Fujian Province (grant numbers 2018J01504, 2018J01505); and the Program for Innovative Research Team in University of Tianjin (grant number TD13-5043). This work is also funded by the College Students Innovation Entrepreneurship Project of Tianjin (201710058076).
